# A quasi-experimental study: cultivating mathematical resilience via intervention in Chinese classrooms

**DOI:** 10.3389/fpsyg.2026.1745726

**Published:** 2026-01-26

**Authors:** Xia Wang, Amalia Madihie

**Affiliations:** 1 Faculty of Cognitive Science and Human Development, Universiti Malaysia Sarawak, Kota Samarahan, Malaysia; 2 Faculty of General Education, An Hui Business and Technology College, Hefei, China

**Keywords:** intervention, mathematical resilience, mathematical resilience scale, quasi-experimental study, Sidek module

## Abstract

**Introduction:**

This study evaluates the efficacy of a culturally adapted Chinese Resilience Intervention Module in enhancing mathematical resilience among university students, grounded in the Sidek resilience framework.

**Methods:**

Mathematical resilience was measured using the validated Chinese Mathematical Resilience Scale. The intervention underwent expert validation for cultural and pedagogical relevance. A quasi-experimental design compared outcomes between intervention and control groups, with a one-month retention test assessing sustainability.

**Results:**

Results demonstrated sustained improvements in resilience scores for the experimental group, persisting through follow-up assessments.

**Discussion:**

The findings support the intervention's potential for identifying at-risk learners and informing targeted support strategies in STEM education. Limitations include geographical specificity to Eastern China and short-term follow-up. This work advances interdisciplinary approaches to resilience-focused pedagogy, advocating for integrated teaching-assessment systems to mitigate mathematical learning barriers.

## 1 Introduction

Mathematics education in China faces significant challenges. Only 1.6% of students gain entry into top-tier universities, approximately 15% enroll in regular universities, and around 60% enter vocational institutions (National Bureau of Statistics of China, 2023). The high-stakes nature of this examination exerts immense pressure on students, exacerbating mathematics anxiety and potentially impeding the development of mathematical resilience (Yang et al., 2023). 21.1% of fourth-grade students and 35.0% of eighth-grade students report experiencing mathematics anxiety, with 69% of middle school students exhibiting moderate to severe levels of anxiety (Xie et al., 2018).

Chinese education system has produced a stark divergence in mathematics learning outcomes. On one hand, Chinese students demonstrate a solid mathematical foundation. Students from economically advanced regions have represented China in the Programme for International Student Assessment (PISA) in 2009, 2012, 2015, and 2018, consistently achieving top rankings in mathematics. Conversely, the emphasis on exam-oriented instruction in China's education system has led to a strong focus on problem-solving drills, often at the expense of creativity and real-world applications. The heavy academic workload, coupled with intense parental and societal expectations for high scores, places considerable stress on students, potentially diminishing their interest and engagement in mathematics. PISA data indicate that Chinese students experience higher levels of mathematics-related anxiety than their global counterparts. Many report feelings of nervousness and lack of confidence in mathematics, which may negatively impact their long-term interest in the subject and their ability to develop mathematical resilience.

Despite the increasing global emphasis on STEM education, research on mathematical resilience in Asian student populations remains limited. This study seeks to bridge this gap by evaluating the validity, reliability, and effectiveness of mathematical resilience interventions.

Current mathematical resilience interventions predominantly operate within school ecosystems, emphasizing classroom architecture, institutional scaffolding, and pedagogical diversification (Jansen et al., 2019). While self-regulated learning frameworks demonstrate academic efficacy, their implementation requires intensification for senior students navigating critical STEM developmental phases (Berger et al., 2020). Contemporary approaches integrate motivational architecture through value-oriented strategies—highlighting intrinsic, achievement, and utility dimensions—to optimize cognitive engagement (Tossavainen et al., 2021). This paradigm necessitates educators' adoption of growth mindsets and instructional alignment with learners' relevance perception (Walton and Yeager, 2020). Nevertheless, such motivation-centric models exhibit inherent constraints: their reliance on variable intrinsic factors and inability to reconfigure extrinsic learning environments challenge long-term sustainability.

Technology-mediated interventions present complementary pathways, with video-based modeling systems proving particularly effective for learners with cognitive barriers (Santagata et al., 2021; Yakubova et al., 2016). Digital pedagogic further augment mathematical cognition through computational problem-solving frameworks and adaptive learning platforms (Li et al., 2020; Videla et al., 2022). However, these innovations introduce systemic risks: infrastructure dependencies exacerbate resource disparities; passive consumption patterns in video instruction reduce formative feedback loops; and technological over reliance may erode interpersonal learning dynamics, potentially widening equity gaps in under-served populations.

Targeted interventions address learning disparities by enhancing problem-solving capacities and mathematical proficiency, yet confront systemic trade-offs: self-regulated learning undervalues collaborative cognition, motivational frameworks exhibit transient efficacy, video-based instruction restricts adaptability, and technology-dependent models amplify resource inequities (Apostolidu and Johnston-Wilder, 2023; Doll and Song, 2023). To reconcile these constraints, we propose the Resilience Intervention Module, anchored in Sidek's tripartite framework, which operationalizes real-world problem translation and meta-cognitive skill development (Sidek and Jamaludin's, 2005). Pre-post assessments via the Mathematical Resilience Scale validate its efficacy in fostering engagement and mitigating gender-based stereotypes through structured pedagogical scaffolding (Donolato et al., 2020).

Globally, resilience integration diverges strategically: U.S. practices emphasize instructional optimization, the U.K. embeds resilience in national curricula, while Singapore prioritizes achievement-oriented pedagogic (Apostolidu and Johnston-Wilder, 2023). Despite these advances, China's unique ecosystem—marked by hyper-competitive academic pressures and collectivist achievement norms—remains under-explored. This modular design bridges this gap by embedding resilience explicitly within curricular architecture, offering a culturally adaptive blueprint. Future iterations must address demographic variability and institutional resource constraints to ensure scalable implementation, leveraging international insights for localized refinement.

A critical application of these mainstream resilience intervention paradigms within educational ecosystems characterized by hyper-competitive examinations, collectivist social norms, and systemic academic pressure—such as that of China—reveals a significant contextual misalignment. Specifically, school-ecosystem approaches, which rely on institutional restructuring and pedagogical diversification, may struggle to create space for systemic innovation in an environment dominated by high-stakes testing and an intensive curricular pace. Self-regulated learning frameworks, while effective in fostering individual agency, often presuppose a degree of learner autonomy and prioritize individual cognition over collective cognition, thereby underestimating the pedagogical potential and cultural resonance of structured, teacher-guided, and peer-supported learning characteristic of Chinese classrooms. Motivation-oriented interventions, designed to enhance intrinsic or utility value, risk being overshadowed by the overwhelming and non-negotiable extrinsic value attributed to the Gaokao. Technology-dependent models may exacerbate pre-existing urban-rural resource disparities and promote passive content consumption, which runs counter to the interactive, discourse-intensive problem-solving practices valued in local pedagogy.

Therefore, the key gap this study seeks to address is not merely geographical—that is, not simply a lack of research on resilience within Chinese populations—but fundamentally conceptual and design-oriented. What is currently missing is an intervention deliberately engineered to operate within and leverage the very structures that often undermine resilience. Rather than attempting to circumvent or dilute these dominant features, our “Resilience Intervention Module” strategically embeds resilience-building mechanisms into them, transforming potential sources of anxiety into structured opportunities for metacognitive development and adaptive coping. This approach aims to establish a culturally congruent pathway to fostering mathematical resilience, one that aligns with, rather than alienates, the existing educational reality.

Although mathematical resilience has been extensively studied as a critical protective factor in Western educational contexts (Johnston-Wilder and Lee, 2010; Sokolowski and Necka, 2016; Silver, 2022), related exploration focusing on Asian student populations remains notably scarce. A systematic review has indicated that most resilience literature is concentrated in Western contexts, making findings difficult to apply in Asian policy and intervention settings (Blessin et al., 2022). Recent empirical research on Chinese senior high school students has also highlighted the relative scarcity of studies examining the status and influencing factors of resilience within this specific population (Supervía et al., 2022). In terms of research depth, the limited existing studies involving Asian students are mostly confined to correlational surveys using cross-cultural scales. There is a serious lack of deeply culturally-adapted intervention designs and mechanism exploration that specifically target the region's unique high-pressure, highly competitive educational ecology. Therefore, this study aims to address not merely a geographical data gap, but a methodological and design philosophy gap: namely, to develop and validate a mathematical resilience intervention that is not added as a standalone curriculum but is deeply embedded within the mainstream pedagogical practices and cultural narratives of the local context.

The intervention utilized in this study is fundamentally shaped by specific cultural and systemic elements unique to China. By embedding resilience strategies within the high-stakes, exam-oriented preparation that characterizes secondary education in China, anxiety-inducing drills are reframed as opportunities for metacognitive growth. Furthermore, cultural factors such as profound societal and parental expectations, as well as the collective orientation toward academic achievement, have likely shaped the module to emphasize group-based problem-solving and value-oriented strategies, aligning familial utility with intrinsic motivation. The design likely integrates the pedagogical strengths of China in solid foundational problem-solving while explicitly addressing its traditional shortcomings in creativity and real-world application. Thus, the distinctiveness of the intervention lies in its deliberate integration of an international resilience framework with the specific pressures, motivational architectures, and curricular realities of the Chinese educational ecosystem, aiming to cultivate resilience from within the very system that often undermines it.

The theoretical underpinnings of this study have been strengthened by the deliberate integration of resilience theory (Masten, 2001) as the principal conceptual framework. This established theory provides the foundational structure for explicitly defining and operationalizing mathematical resilience, which in turn informs the core design of our intervention. Subsequently, this theoretical lens will be employed to explicate the underlying mechanisms of the intervention and to interpret the resultant findings and their broader implications.

The following hypothesis was formulated:

H1. The Chinese Mathematical Resilience Intervention demonstrates statistically acceptable reliability and validity.
H2. There is no significant difference on mathematical resilience at pre-test and post-test among Chinese students in intervention groups and experimental groups.

## 2 Method

### 2.1 Participants

Participants in this study (*N* = 80) ranged in age from 18 to 20 years. Women (*n* = 46) comprised 57.5% of the sample. Participants were recruited from a college in the Eastern China. This study adhered to the APA's code of conduct regarding treatment of human participants and was approved by the Universiti Malaysia Sarawak andAnhui Business and Technology College. Mathematical resilience levels were assessed at three intervals: pre-intervention, post-intervention and one-month post-intervention.

### 2.2 Procedure

The Chinese Mathematical Resilience Intervention was implemented as a 12-week program comprising 12 thematic modules. Targeting first-year STEM undergraduates with foundational mathematical competency, the intervention integrated resilience-building strategies into the standard calculus curriculum. A quasi-experimental pretest-posttest design with a control group was adopted (Table 1).

**Table 1:** Diagram of experiment study and pre-test and post-test with the control design.

**Pre-test**	**Group**	**Independent variable**	**Post-test**	**Retention test**
O1	Intervention group	Intervention	O2	O3
O1	Control group	No-intervention	O2	O3

Oi (*i*: 1,2,3): assessment of the intervention group and control group.

Content validity was established via Delphi review with three mathematics education experts and two psychometric specialists, achieving a Content Validity Index of 0.89. Reliability testing with 20 pilot participants demonstrated strong internal consistency (Cronbach's α = 0.81–0.84 across subscales).

The Mathematics Resilience Scale was administered at all three time points. Baseline equivalence between intervention group and control group was confirmed through independent *t*-tests (*p* > 0.05 for all pretest measures). SPSS 26.0 facilitated hypothesis testing via Repeated-measures ANOVA for longitudinal resilience trajectories, Bonferroni-adjusted pairwise comparisons between assessment phases, Cohen's d calculations for effect size quantification, Hierarchical linear modeling to account for classroom-level clustering.

### 2.3 Materials

The *Mathematics Resilience Scale* (Kooken et al., 2016) assessed four dimensions of mathematical engagement using 24 items on a 7-point Likert scale (1 = *strongly disagree*, 7 = *strongly agree*). Sub scales included: Value, Struggle, Growth, and Resilience. Total scores ranged from 24 to 168, with higher scores indicating stronger resilience. Original validation studies reported strong internal consistency (α = 0.83–0.91 across sub scales) and construct validity via confirmatory factor analysis (CFI = 0.93, RMSEA = 0.06) (Kooken et al., 2016). In this study, Cronbach's α coefficients of the Chinese version of the mathematical resilience scale (Wang et al., 2025) ranged from 0.79 (Struggle) to 0.88 (Resilience).

## 3 Results

Consistent with H1, the Chinese Mathematical Resilience Intervention demonstrated strong content validity. Lawshe's Content Validity Ratio analysis yielded unanimous expert agreement (CVR = 1.0 for all items), exceeding the critical threshold for three evaluators (Lawshe, 1976). Student feedback further supported feasibility, with 70.62% of responses meeting the positive reception criterion (Percentage of Positive Responses ≥ 50%), a benchmark for intervention acceptability (Bowen et al., 2009). As hypothesized, the Chinese Mathematical Resilience Intervention exhibited excellent internal consistency across all themes. Cronbach's α coefficients ranged from 0.951 to 0.988, substantially exceeding the 0.70 acceptability threshold for research instruments (Nunnally and Bernstein, 1994). These results confirm H1 regarding the module's psychometric stability.

Prior to intervention implementation, preliminary analyses were conducted to ensure baseline equivalence in dependent variable mean scores between experimental and control groups during the pretest phase. Initial group comparisons employed one-way analysis of variance (ANOVA) to examine mean score differences between the intervention and control cohorts.

The analytical framework incorporated Levene's test of variance homogeneity to verify the stability of dispersion patterns in quantitative measures across experimental groups. Interpretation criteria followed standard conventions: a statistically significant result (*p* < 0.05) indicated heteroscedasticity between groups, while non-significance (*p* > 0.05) confirmed variance homogeneity.

Subsequent analysis utilized a two-way independent ANOVA design with *post-hoc* multiple comparison adjustments to delineate inter-group mean score differences. For multivariate normality assessment in MANOVA procedures, Box's *M* test was applied to evaluate the equality of covariance matrices across study conditions. Then, a repeated measures ANOVA was employed to assess the changes in the intervention and control groups across different time points. All empirical results from these preliminary analyses are systematically presented in Table 2.

**Table 2:** Preliminary analyses.

**Sub-scale**	**Value**	**Struggle**	**Growth**	**Resilience**
Mean square	251.222	592.922	4.41	295.84
*F*	5.998	21.139	1.233	9.232
*P*	0.015^*^	0.000^**^	0.125	0.003^**^
Mean difference	1.585	2.435	−0.75	1.72
Standard error	0.647	0.53	0.299	0.566
*t*-value	2.449	4.598	−2.509	3.038
Welch *F*	5.931	21.261	1.645	9.314
Generalized eta-squared	0.015	0.042	0.005	0.023
Partial η2	0.069	0.187	0.021	0.105

^*^p < 0.05 ^**^ *p* < 0.01.

Given that the sample size was below 50 in all instances, the Shapiro-Wilk (S-W) test was applied, for groups with *p*-values greater than 0.05, the *t*-test was deemed appropriate. The results are showed in Table 3.

**Table 3:** The results of the shapiro-wilk test and *t*-test.

**Sub-scale**	**Comparison**	**Shapiro-wilk test**		**Mean difference**	**SD of difference**	**Cohen's d**	***t***
		***W***	***P***				
Value	Pre-post	0.951	0.085	−4.45	10.061	0.442	−2.797
	Post -retention	0.968	0.3	−2.9	9.378	0.309	−1.956
Struggle	Pre-post	0.958	0.144	−0.63	8.877	0.07	−0.445
	Post -retention	0.959	0.15	−0.95	9.131	0.104	−0.658
Growth	Pre-post	0.973	0.46	−1	3.013	0.332	−2.099
	Post -retention	0.958	0.145	−0.13	2.388	0.052	−0.331
Resilience	Pre-post	0.957	0.135	−1.68	7.577	0.221	−1.398
	Post -retention	0.963	0.214	−1.95	7.88	0.247	−1.565

## 4 Discussion

This study tested hypotheses proposing that the Chinese Resilience Intervention would significantly enhance mathematics resilience compared to the control group, and the intervention's effects would vary over time. Both hypotheses were partially confirmed. A significant main effect of the intervention on Mathematical resilience was observed (*p* < 0.001), with *post-hoc* comparisons confirming stronger resilience gains in the experimental group (*p* = 0.015). These results imply that the intervention effectively improved Mathematical resilience, but its impact remained relatively stable across assessment periods rather than fluctuating systematically.

The findings underscore the efficacy of structured interventions in fostering academic resilience, aligning with broader research on resilience-building programs (Dray et al., 2017). The medium effect size (partial η^2^ ≈ 0.06) aligns with Cohen's (2013) benchmarks for education-focused interventions, suggesting practical relevance. The non-significant time effect, however, implies that resilience gains may plateau shortly after intervention delivery, a pattern noted in some school-based interventions and highlighting the need for reinforcement strategies (Durlak et al., 2011).

The intervention is a culturally adaptive instrument designed to transform core characteristics of mathematics education in China. It systematically integrates components of growth mindset training (Dweck, 2006) and metacognitive reflection (Flavell, 1979) into high-intensity practice. This approach follows best practices for cultural adaptation, which emphasize deeper contextual integration over superficial translation (Barrera and Castro, 2006; Bernal et al., 2009). Unlike common approaches in Western models, which often aim to replace intensive skill training or diminish competitiveness, this intervention acknowledges and embraces the central role and practical necessity of high-intensity practice and examination-oriented preparation within Chinese educational ecosystem. Consequently, our goal is not to weaken or replace this characteristic but to systematically integrate components of growth mindset training and metacognitive reflection into it, thereby transforming its intrinsic processes and the students' experiential essence.

Specifically, the intervention shifts the focus from the “mere pursuit of problem-solving accuracy and speed” to the simultaneous cultivation, during the problem-solving process, of cognitive restructuring in the face of setbacks, monitoring and adjustment of strategies, and a deep-seated identification with the value of learning. This repositions resilience from an external, supplementary psychological concept into an internalized, operational capability and tool for students to navigate the existing high-pressure academic environment. It assists students in transforming external pressures and personal anxieties into manageable metacognitive challenges oriented toward mastery and understanding. This design directly addresses the unique stressors within the Chinese context and seeks to carve out a learning space characterized by greater agency and psychological elasticity for students within these structural constraints.

Therefore, this intervention does not presuppose fundamental changes to educational culture or structure. Instead, it strives to empower students, enabling them to develop a healthier, more sustainable, and equally effective way of running within the existing and challenging academic track. Future research and practice should further explore how to scale and institutionalize this “embedded” model of resilience cultivation across diverse regions and school resource contexts.

The cultural adaptation is a systematic re-conceptualization. We strive to integrate core strategies into existing instructional routines to ensure feasibility and high implementation fidelity—a critical factor for intervention success (Durlak and DuPre, 2008). The observed effects are likely attributable to these specific adaptation mechanisms, particularly the integration of individual and collective-level dynamics.

This approach ensures the intervention's feasibility and acceptability, prevents additional burdens on teachers and students, and thereby secures a high degree of implementation fidelity. It also minimizes student unfamiliarity and resistance, safeguarding the intervention's ecological validity. The observed intervention effects are likely attributable not to the abstract resilience theory per, but precisely to these specific mechanisms of cultural adaptation. The key mechanism of effectiveness may lie in its successful integration of individual-level approaches to viewing mistakes with collective-level dynamics of mutual assistance and shared progress.

This study demonstrates that effective cultural adaptation should target deeper motivational systems. It suggests future interventions should analyze the target culture's core practices and social structures, as advocated by ecological models of intervention (Bronfenbrenner, 1979). It suggests that future research, when designing interventions, should prioritize the analysis of the target educational culture's core instructional practices, dominant value discourses, and social interaction structures, and proceed with targeted design accordingly.

This study is subject to several important limitations. First, the participant sample was geographically homogeneous, consisting of university students from a single academic context in China, which constrains the generalizability of the findings. Second, the primary outcome of mathematical resilience was assessed solely through self-report measures, which may introduce social desirability bias and may not fully capture behavioral expressions of resilience. Third, the 12-week intervention period lacked a long-term follow-up assessment, limiting our understanding of the durability of the observed resilience gains. Finally, the intervention's deep cultural adaptation, while a core strength, inherently bounds its immediate applicability to the specific, high-pressure context of Chinese mathematics education from which it was designed.

These limitations chart a clear course for subsequent research. To address concerns of generalizability, future studies should replicate the intervention with more diverse populations, including students of different age groups (e.g., secondary school), from varied socioeconomic backgrounds, and across distinct cultural regions. To overcome methodological constraints, researchers should incorporate multi-method assessments, such as behavioral observations of persistence during challenging tasks or teacher evaluations of student engagement, to triangulate self-report data. To evaluate the sustainability of effects, longitudinal designs with follow-up assessments at 6 and 12 months post-intervention are essential. Finally, to explore the transferability of the approach, the core meta-design process—embedding resilience-building into mainstream pedagogical routines—should be piloted in other educational contexts, guided by established frameworks for principled cultural adaptation. We now explicitly frame the study as “an open proof-of-concept” that opens several defined avenues for development rather than presenting a closed finding. The final paragraph underscores that addressing these limitations is crucial for advancing the field.

Based on the findings within the Chinese context, a pivotal question arises regarding the broader applicability of this intervention model. We posit that the core design principle underpinning the module—reframing resilience-building as an integrated process embedded within mainstream, culturally-validated pedagogical practices, rather than implementing it as an add-on or standalone program—holds significant transferable value for other educational systems. This principle shifts the focus from importing external content to strategically enhancing meta-cognition within existing, high-frequency instructional routines.

However, the transfer of this approach is not a matter of direct replication but one of principled adaptation. The generalizable element lies primarily in the meta-design process that guided our development. This process mainly involves the direct integration of meta-cognitive reflection, cognitive reappraisal, and value-reframing strategies into the implementation of the intervention. It offers a replicable heuristic for educators and researchers working in other educational contexts characterized by strong institutionalized traditions and performance pressure.

Therefore, the contribution of this study is not to provide a universal blueprint for resilience intervention but to serve as a proof-of-concept for a situated, culturally-responsive design philosophy in educational psychology. Its ultimate utility in other contexts depends critically on the work of re-contextualization—a careful analysis of local pedagogical realities and the subsequent design of integrated solutions that are both psychologically sound and culturally congruent. Future research should systematically pilot this meta-design process in diverse settings to refine its principles and delineate the boundary conditions of its effectiveness, thereby advancing a more nuanced and context-sensitive science of resilience education.

## Data Availability

The raw data supporting the conclusions of this article will be made available by the authors, without undue reservation.
