# Supplementary material for: A quasi-experimental study: cultivating mathematical resilience via intervention in Chinese classrooms
Source: Front Psychol. 2026 Jan 26;17:1745726. doi: 10.3389/fpsyg.2026.1745726 (PMC12883801; doi:10.3389/fpsyg.2026.1745726)
Supplement: Supplementary file 1 [file Supplementary_file_1.docx]

**APPENDIX**

**Mathematical resilience Scale**

Part A

| Your gender |  |
| --- | --- |
| Are you a university student? |  |
| Your age |  |
| The geographic location of your home is: |  |
| Are you an only child? |  |
| What was your most recent mathematics exam score？ |  |
| Your major: |  |

Part B

| **No.** | **Item** | **Strongly Disagree -Strongly Agree** |
| --- | --- | --- |
| 1 | Mathematics is essential for my future. | 1 2 3 4 5 |
| 2 | Mathematics will be useful to me in my life’s work. | 1 2 3 4 5 |
| 3 | Mathematics courses are very helpful no matter what I decide to study. | 1 2 3 4 5 |
| 4 | Knowing mathematics contributes greatly to achieving my goals. | 1 2 3 4 5 |
| 5 | Having a solid knowledge of mathematics helps me understand more complex topics in my field of study. | 1 2 3 4 5 |
| 6 | People who are good at mathematics have more opportunities than those who aren’t good at math. | 1 2 3 4 5 |
| 7 | Thinking mathematically can help me with things that matter to me. | 1 2 3 4 5 |
| 8 | It would be difficult to succeed in life without mathematics. | 1 2 3 4 5 |
| 9 | Mathematics develops good thinking skills that are necessary to succeed in any career. | 1 2 3 4 5 |
| 10 | Everyone struggles with mathematics at some point. | 1 2 3 4 5 |
| 11 | Good mathematicians experience difficulties when solving problems. | 1 2 3 4 5 |
| 12 | Successful people who work in math-related fields struggle when working on hard mathematics problems. | 1 2 3 4 5 |
| 13 | Everyone makes mistakes at times when doing mathematics. | 1 2 3 4 5 |
| 14 | Struggle is a normal part of working on mathematics. | 1 2 3 4 5 |
| 15 | People in my peer group struggle sometimes with mathematics. | 1 2 3 4 5 |
| 16 | People who are good at mathematics may fail a hard mathematics test. | 1 2 3 4 5 |
| 17 | Mathematics teachers have difficulties sometimes when answering a mathematics question. | 1 2 3 4 5 |
| 18 | Everyone can get better at mathematics if they try. | 1 2 3 4 5 |
| 19 | Mathematics can be learned by anyone. | 1 2 3 4 5 |
| 20 | If someone is not a mathematics person, they won’t be able to learn much mathematics. | 1 2 3 4 5 |
| 21 | If someone is not good at mathematics, there is nothing that can be done to change that. | 1 2 3 4 5 |
| 22 | People are either good at mathematics or they aren’t. | 1 2 3 4 5 |
| 23 | I believe a person’s mathematics ability is determined at birth. | 1 2 3 4 5 |
| 24 | Some people cannot learn mathematics. | 1 2 3 4 5 |
| 25 | I believe I can grow in my knowledge of mathematics. | 1 2 3 4 5 |
| 26 | When I have done poorly on something related to mathematics, I know how to adapt. | 1 2 3 4 5 |
| 27 | I sometimes get discouraged by difficulties in mathematics, but I bounce back. | 1 2 3 4 5 |
| 28 | I have strategies to use when I get stuck trying to solve mathematics problems. | 1 2 3 4 5 |
| 29 | When I fail or do poorly on a mathematics test, I know I have to work harder. | 1 2 3 4 5 |
| 30 | When I struggle with mathematics, I return to it until I get it. | 1 2 3 4 5 |
| 31 | When I experience a setback in something related to mathematics, I seek encouragement from others. | 1 2 3 4 5 |
| 32 | I sometimes find mathematics confusing, but I stick with it. | 1 2 3 4 5 |
| 33 | When I don’t do as well as I hoped on a mathematics task or test, I keep trying until I can do it. | 1 2 3 4 5 |

**Introduction of the modular**

**Rational:**

Calculus is a fundamental and crucial component of the field of mathematics. It serves as a tool for describing and analyzing changes, explaining various phenomena in nature, society, and science, and solving practical problems. Widely applied in fields such as physics, engineering, economics, and biology, calculus provides a profound understanding of the changes in functions and curves and the reasons behind these changes. It forms the cornerstone of mathematical analysis, introducing essential concepts like limits, derivatives, and integrals, which are foundational for advanced mathematical disciplines such as differential equations, real analysis, and complex analysis. Moreover, calculus serves as the basis for many advanced disciplines, including physics, engineering, computer science, and economics. Learning calculus establishes a solid mathematical foundation for further exploration in these fields. The study of calculus requires deep abstract thinking and problem-solving skills, qualities essential for various disciplines and professions.

**Modular instruction:**

The CRI-MR(Calculus Readiness Initiative) is divided into 12 units, to be completed over 12 weeks. It covers the main concepts of calculus, including The concept of derivative, Computation of derivatives, Applications of derivatives, The concept of differentiation, Application of differentiation, Concept of Antiderivative, Calculation of Antiderivative, The Concept of Definite Integral, Calculation of definite integral, and Application of Definite Integral. Each module is structured with headings, instruments/tools, time, objectives, rationale, steps, discussion, conclusion, and appendix.

**Purpose:**

The CMR Iis tailored for students with a strong foundation in mathematics, both at the high school and university levels. It caters to professionals in various scientific, engineering, economic, and social science fields who need to apply mathematical tools in their respective domains. While calculus is a core course for students majoring in science and mathematics, it also serves as an elective or prerequisite course for other majors. Typically introduced in the first or second year of study, calculus provides students with a deep mathematical foundation for subsequent specialized courses. Given its widespread applications, calculus is also an integral part of university courses in engineering, physics, computer science, economics, and other related fields.

**Objectives:**

Upon completion of the CRI, students should be able to calculate limits and understand their applications in function continuity and derivatives. They should be proficient in computing derivatives, understanding their geometric and physical significance, and applying derivatives to solve real-world problems. Students should grasp the concept of differentiation and use it to characterize local changes in functions. Additionally, they should be capable of computing indefinite integrals, understanding the geometric and physical meanings of integrals, and applying integrals to solve problems related to area, volume, and cumulative rates of change. Understanding the fundamental theorems of calculus, such as the Newton-Leibniz formula, and applying them in integral computations, recognizing the relationship between integrals and derivatives, and applying calculus concepts and skills to solve real-world problems in various fields are also expected outcomes. The overall goal is to cultivate abstract thinking, logical reasoning, and problem-solving skills in students.

**Prerequisites:**

Students need to possess a solid foundation in algebra, including solving equations, polynomial operations, factoring, and square root operations. A familiarity with trigonometry, especially involving angles, trigonometric functions, and trigonometric identities, is beneficial. Understanding basic function concepts, such as graphing functions, properties of functions, composite functions, and inverse functions, is essential. Knowledge of elementary functions (such as polynomial functions, exponential functions, logarithmic functions, and trigonometric functions) is important. Some understanding of mathematical analysis, especially basic concepts related to limits, is helpful. Familiarity with the concepts of infinitesimals and infinities, as well as an understanding of sequences and series' basic properties, forms the foundation for learning calculus. A grasp of basic geometry concepts, particularly in plane geometry and coordinate geometry, enhances comprehension of the geometric aspects of calculus.

**Teaching Objectives:**

The instructional goals of calculus are aimed at developing a profound understanding of calculus concepts and skills in students and enabling them to apply this knowledge to real-world problems. The teaching of calculus should foster abstract thinking, logical reasoning, and problem-solving skills. It should empower students not only to acquire knowledge but also to apply that knowledge flexibly to various mathematical and practical problems. Ultimately, students should be able to use calculus in mathematical modeling, transforming real-world problems into mathematical problems and employing calculus methods for modeling and analysis.

**Testing:**

This involves conducting MRS (Midterm Review Session) tests and assessments for learners at the end of each module. Students will engage in self-assessment through the completion of self-evaluation forms, standardized academic achievement tests, and evaluations to assess their performance in achieving the module objectives.

**EXAMPLIE MODULAR**

**Title** Concept of Derivatives

**Time** 2 classes, 160 min

**Applicable objects** Freshmen

**Instruments/tools**

Pre-test, post-test, MRS, Video, Slide, text book,

**Objectives**

Understand the concepts of average rate of change and instantaneous rate of change; grasp the relationship between the average rate of change and the instantaneous rate of change. Understand the practical background of the concept of derivatives and appreciate the thoughts and meanings behind derivatives.

Stimulate interest in learning through an understanding of the history and practical applications of derivatives; experience and identify with the dialectical perspective of unity in opposition between finite and infinite, accepting the use of dialectical materialism in dealing with mathematical problems involving dynamic changes.

Cultivate students' abilities to compare, analyze, and generalize through solving practical problems.

**Rational**

Applying a cooperative problem-oriented learning approach to organize teaching, the learning process is designed as group activities where students collaborate to solve problems. By understanding the history of mathematical development and appreciating the value of calculus, students experience the twists and turns in the development of differential concepts, gaining insight into the struggles of learning mathematical concepts. Through group discussions, sharing ideas, and integrating viewpoints, students' interest and initiative are stimulated.

Within the group, students have more autonomy, deciding how to organize work, allocate tasks, and find solutions suitable for their team. This helps cultivate students' self-directed learning abilities. Through group collaboration, students not only acquire subject knowledge but also develop teamwork skills, including effective communication, listening, task delegation, and conflict resolution. Encouraging students to think, question, and analyse during the problem-solving process fosters critical thinking and problem-solving skills.

**Steps**

Pre-class:

1. Complete the first round of data collection for MRS.

2. Before the class, assign pre-reading tasks by dividing students into 8 small groups. Assign learning tasks related to the origin of the concept of derivatives. Explore the introduction of derivatives stemming from the study of limits. Discuss the differences and connections between Newton's fluxions and Leibniz's calculus concepts. Conduct research on the history of mathematical development, and have groups report their findings.

In-class:

1. Evaluate and provide feedback on each group's presentation of their learning outcomes.

In 1687, Newton introduced the concept of derivatives in his work "Mathematical Principles of Natural Philosophy" to describe the instantaneous velocity of variable linear motion. Newton considered the case of an object moving in a straight line, defining instantaneous velocity as the object's velocity at a specific moment, i.e., the ratio of the distance moved in an infinitely small-time interval to the time interval. If the position of the object is described as a function of time, then the instantaneous velocity is the derivative of the position function with respect to time.

Leibniz, another founder of calculus, independently introduced the concept of derivatives. In the late 17th century, Leibniz independently developed calculus simultaneously with Newton. When introducing the concept of derivatives, Leibniz focused on the tangent line problem at a point on a plane curve. He considered a point on the curve, constructed a line connecting this point with another point on the curve, and created a tangent line approaching the curve. Leibniz defined the slope of the tangent line (i.e., the degree of inclination) as the rate of change between two points on the curve.

By telling the stories of these two mathematicians, students can understand the inevitability and twists in the development of calculus, recognize the value of mathematics, and spark interest.

2. Teacher Summary

Introduce the concept of derivatives through analysing the rate of change in graphs.


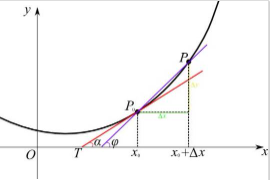

$$f'(x_{0})=\lim_{\Delta x\to0} \frac{\Delta y}{\Delta x}=\lim_{\Delta x\to0} \frac{f(x_{0}+\Delta x)-f(x_{0})}{\Delta x}$$

3. Student Task 1

Explore the relationship between limits and rates of change, specifically analyzing the definition of derivatives with examples of power functions, exponential functions, and trigonometric functions. Students are grouped for discussion, encouraged to raise questions, and the teacher collects these questions for summarization.

4. Teacher Summary

There is a close relationship between limits and rates of change, especially in calculus. Limits are a mathematical concept used to describe the behaviour or variation of a function as a variable approaches a specific value. Rate of change, on the other hand, describes the instantaneous change of a function at a particular point. In calculus, derivatives represent a special form of rate of change, with limits serving as the foundation for calculating derivatives.

5. Using a linear function as an example, analyse and explain the definition of derivatives and their relationship with limits.

6. Student Task 2

Using power functions

$y=x^{2}$, $y=x^{3}$,$y=x^{n}$

as examples, engage in group discussions on how to utilize the definition of derivatives to calculate them, explore patterns by combining previously learned knowledge with recently acquired knowledge.

7. Teacher Summary:

Three Steps to Derivation

According to the definition of derivatives, the process generally involves the following three steps.

Find the increment of the function:

$$\Delta y=f(x+\Delta x)-f(x);$$

Determine the ratio of the two increments:

$$\frac{\Delta y}{\Delta x}=\frac{f(x+\Delta x)-f(x)}{\Delta x}$$

Evaluate the limit:

$$y'=\lim_{\Delta x\to0} \frac{\Delta y}{\Delta x}.$$

8. Student Individual Practice

Use the definition of derivatives to find the following limits (assuming each limit exists).

(1) $\lim_{x\to a} \frac{f(2x)-f(2a)}{x-a};$

(2) $\lim_{x\to0} \frac{f(x)}{x},$ where$f(0)=0.$

9. Students Discussion

Students present their answers and analyse and discuss challenging points, such as algebraic simplification, which may involve complex algebraic operations like factoring and combining like terms. It may also require simplification or substitution to handle limits where the denominator approaches zero. Difficulties may arise when the function is discontinuous or the derivative is non-existent, requiring additional analysis.

10. Student Task Three:

Group discussion on finding the derivative at x=0 for y=|x|.

11.Discuss and analyse the answers

The derivative of y=|x| at x=0 does not exist because, according to the definition of derivatives, the limit at this point does not exist. The teacher introduces the concepts of left and right derivatives.

$$y_{+}^{'}\left( x_{0} \right)=\lim_{x\to0+} \frac{f\left( x \right)-f\left( x_{0} \right)}{x-x_{0}}$$

$$y_{-}^{'}\left( x_{0} \right)=\lim_{x\to0-} \frac{f\left( x \right)-f\left( x_{0} \right)}{x-x_{0}}$$

Students transition from the concepts of left and right limits to the concepts of left and right derivatives.

12. Student Task 4

Group discussion on categorizing cases where derivatives do not exist and providing examples.

13.Teacher summarizes student answers

Derivatives may not exist when left and right derivatives are not equal, or at least one of them is non-existent.

14. Classroom Task 5

Group discussion on identifying functions that may have non-existent derivatives, considering composite functions and piecewise functions.

For example: y$=ⅇ^{\frac{1}{x}}$ , where x=0

$y=\left\{ \begin{aligned} x, x>1 \\ 2, x\leq1 \end{aligned} \right.$ , where x=1

15. Teacher summarizes student responses.

When the left derivative and the right derivative are not equal, or at least one of them does not exist, the derivative does not exist.

16. Student Task 6

Group discussion on the relationship between the continuity and differentiability of functions, completing the transfer of concepts. Explore the characteristics of continuous and differentiable functions, as well as functions that are continuous but not differentiable.

17. Teacher Summary

Derivability implies continuity, but continuity does not necessarily imply differentiability.

18. Individual Assignment

Short paper: The Meaning and Practical Value of the Concept of Derivatives, with 20 accompanying exercises.

Group Assignment

Preparatory homework for the next class.

**Discussion:**

Understanding the history of calculus allows for a deeper comprehension of the origins and development of related concepts. This aids in understanding the overall trajectory of mathematical development, integrating calculus into the entire mathematical system, and grasping the intuition and thought processes behind the concepts. Additionally, it can spark learners' interest in the subject by connecting calculus development to real-world problem-solving, emphasizing its practicality and importance. Exploring the relationship between limits and derivatives requires students to analyze mathematical concepts deeply, understand their underlying principles, and articulate mathematical phenomena clearly, fostering a profound understanding and expression of mathematical problems.

The transfer of knowledge from left and right limits to left and right derivatives enables students to apply previously learned concepts and skills to solve new problems. Transferable mathematical knowledge stimulates creative and critical thinking, allowing students to apply mathematical knowledge to new areas or problems. It requires students not only to master surface-level knowledge but also to understand the principles and concepts behind it. This encourages the development of critical thinking, the ability to analyse problems deeply, and the need for creative thinking to discover solutions and evaluate their effectiveness. The transfer of mathematical knowledge also demands adaptability to different contexts and application areas.

Analysing functions where derivatives do not exist requires students to analyse the characteristics and properties of functions without derivatives, understanding why derivatives do not exist at certain points. This cultivates students' ability to analyse and reason about mathematical phenomena deeply. Studying functions with non-existent derivatives also helps students develop an intuitive sense of function graphs and changes. Critical thinking is required to evaluate the impact of non-existent derivatives on mathematical and real-world problems, contributing to the development of students' critical thinking and judgment. Logical thinking is also essential for understanding the conditions and limitations of non-existent derivatives.

Discussions on the relationship between derivatives and continuity require students to understand and apply these concepts to specific functions. This helps develop students' understanding and application of abstract concepts. Discussing the relationship between continuity and differentiability also demands logical thinking, analyzing conditions, reasoning conclusions, and understanding the logical relationship between these two concepts. In group discussions, students need to express their viewpoints, reasoning, and conclusions clearly, enhancing their mathematical communication skills and promoting mutual inspiration among students.

Through these discussions, students can better appreciate the interesting aspects of the subject, making learning more engaging and meaningful. By exploring the history, analyzing concepts, and discussing relationships, students can deepen their understanding of calculus, fostering critical thinking, logical reasoning, and effective communication in the realm of mathematics.

**Conclusion**

The discussion emphasizes the importance of delving into the history of calculus to deepen understanding of related concepts, integrate calculus into a broader mathematical framework, and ignite learners' interest. Exploring the relationship between limits and derivatives involves in-depth analysis of mathematical concepts, fostering students' profound understanding and expressive abilities. The transfer of knowledge from left and right limits to left and right derivatives enables students to apply concepts and skills to latest problems, promoting creativity, critical thinking, and adaptability. Analyzing functions where derivatives do not exist cultivates students' analytical and reasoning abilities. Discussing the relationship between derivatives and continuity promotes understanding and application of abstract concepts, encouraging logical thinking and clear expression in group discussions. Overall, these approaches contribute to a comprehensive understanding of calculus, enhancing students' mathematical skills and fostering creativity, critical thinking, and effective communication, allowing students to experience the inevitable setbacks and struggles in learning mathematics. Through communication and discussion, they recognize the possibility of progress in learning, sparking interest, and strengthening mathematical resilience.
